# Maternal–Fetal Implications of Herpes Virus Infection: An Updated Review

**DOI:** 10.3390/diagnostics16081147

**Published:** 2026-04-13

**Authors:** Stefany Silva Pereira, Beatriz Bussi Rosolen, Talita Almeida Durães, Marcela Fermoselle de Vita Silva, Giovanna Alves de Britto, Camila Silva Belo, Thamy Cristina Campos, Gustavo Yano Callado, Susana Cristina Aidé Viviani Fialho, Antonio Braga, Edward Araujo Júnior

**Affiliations:** 1 Discipline of Woman Health, Municipal University of São Caetano do Sul (USCS), São Caetano do Sul 09521-160, SP, Brazil; stefany.pereira@uscsonline.com.br (S.S.P.); beatriz.rosolen@uscsonline.com.br (B.B.R.); talita.duraes@uscsonline.com.br (T.A.D.); marcela.silva1@uscsonline.com.br (M.F.d.V.S.); giovanna.britto@uscsonline.com.br (G.A.d.B.); camila.belo@uscsonline.com.br (C.S.B.); thamy.campos@uscsonline.com.br (T.C.C.); araujojred@terra.com.br (E.A.J.); 2 Albert Einstein Israelite College of Health Sciences (FICSAE), Albert Einstein Israelite Hospital, São Paulo 05652-900, SP, Brazil; gycallado@gmail.com; 3 Department of Maternal and Child Health, School of Medicine, Fluminense Federal University (UFF), Niterói 24070-090, RJ, Brazil; susanaaide@id.uff.br; 4 Department of Gynecology and Obstetrics, School of Medicine, Federal University of Rio de Janeiro (UFRJ), Rio de Janeiro 22240-003, RJ, Brazil; 5 Department of General and Specialized Surgery, School of Medicine and Surgery, Federal University of the State of Rio de Janeiro (UNIRIO), Rio de Janeiro 20270-330, RJ, Brazil; 6 Postgraduate Program in Applied Health Sciences, University of Vassouras (Univassouras), Vassouras 27700-000, RJ, Brazil; 7 Antônio Braga, Maternity School, Federal University of Rio de Janeiro, Rua das Laranjeiras, No. 180, Laranjeiras, Rio de Janeiro 22240-003, RJ, Brazil; 8 Department of Obstetrics, Paulista School of Medicine, Federal University of São Paulo (EPM-UNIFESP), São Paulo 04023-062, SP, Brazil

**Keywords:** herpes simplex virus, diagnosis, pregnancy, vertical transmission, perinatal outcomes, treatment

## Abstract

Herpes simplex virus (HSV) infection is highly prevalent worldwide and poses important risks during pregnancy due to the potential for vertical transmission and severe neonatal disease. HSV-1 is traditionally associated with orofacial lesions and HSV-2 with genital infection; however, HSV-1 has emerged as a significant cause of genital and neonatal herpes. Physiological immunomodulation during pregnancy may facilitate viral reactivation and replication. Vertical transmission may occur intrauterinely, intrapartum, or postnatally, with approximately 85% of neonatal infections acquired during delivery through contact with infected genital secretions. The risk is highest when primary maternal infection occurs in the third trimester, before adequate transplacental transfer of protective antibodies. Neonatal infection may present as disease limited to the skin, eyes, and mouth; central nervous system involvement; or disseminated multiorgan disease, the latter associated with high morbidity and mortality. Maternal infection ranges from asymptomatic viral shedding to painful vesiculoulcerative lesions and, rarely, disseminated disease. Because asymptomatic shedding is common, diagnosis relies on laboratory confirmation using polymerase chain reaction (PCR) or viral culture, with type-specific serology aiding in distinguishing primary from recurrent infection. Management aims to reduce symptoms, viral shedding, recurrences near delivery, and vertical transmission. Acyclovir and valacyclovir are safe and effective in pregnancy. Suppressive therapy from 36 weeks’ gestation reduces recurrences and viral shedding at delivery and decreases the need for cesarean delivery, which is recommended when active lesions or prodromal symptoms are present at labor. Neonatal herpes requires prompt recognition and intravenous acyclovir therapy to reduce mortality and neurological sequelae. Preventive strategies include counseling, behavioral risk reduction, suppressive antiviral therapy, and avoidance of neonatal exposure to active lesions.

## 1. Introduction

Herpes simplex (HSV) is an enveloped DNA virus of the Herpesviridae family and represents one of the most prevalent sexually transmitted infections in the population [1]. It is transmitted from person to person through direct contact of the virus with the mucosa or injured skin. Infections are usually asymptomatic, mild, and nonspecific; the virus manifests through skin lesions, which may be found in the form of grouped erythematous vesicles that can evolve into shallow ulcers with accumulation of dry serous exudate during the healing process of the condition. These lesions are painful and may be accompanied by tingling, burning, stinging, pruritus, regional lymphadenopathy, and fever, malaise, and myalgia in primary infection [2]. HSV type 1 is more prevalent in orofacial lesions and typically resides in the trigeminal ganglia, whereas HSV type 2 manifests more frequently in genital lesions and resides in the lumbosacral ganglia. The virus remains latent and may manifest at other times in the carrier’s life without major repercussions. However, in the gestational context, it has great relevance for fetal and neonatal health [1,3].

The risk of vertical transmission alters the obstetric management of the pregnant woman, as the disease may be transmitted in different ways. Although rare, transplacental or intrauterine transmission represents 5% of cases; in this situation, the fetus may present growth restriction, fetal hydrops, preterm birth, and other common obstetric complications. However, manifestations usually occur within the first 48 h of life with the “classic triad” of cutaneous signs, ocular damage, and central nervous system manifestations. Severity occurs due to the absence of antibodies at the time the disease manifests and is associated with severe maternal disease, especially in primary infection [4].

Postnatal infection accounts for 10% of cases; in this situation, HSV is acquired by the newborn through direct contact with relatives or caregivers infected with labial lesions or mammary lesions in the case of breastfeeding. A severe condition may develop in children whose mothers are seronegative for herpes simplex, as they do not receive antibodies through passive immunity. Finally, the most prevalent type of HSV infection in newborns occurs during the intrapartum period. It occurs during birth, when the newborn encounters contaminated maternal secretions. The disease may be transmitted by symptomatic and asymptomatic women and is more frequent in pregnant women with primary infection [4].

Regardless of the route of infection, neonates are classified according to the clinical manifestations of the disease as: (a) Skin-Eye-Mouth (SEM) disease, in which the newborn presents cutaneous, ocular, or oral manifestations; (b) disease localized to the central nervous system; and (c) the worst prognosis, with disseminated disease involving all systems. SEM disease and the disseminated form usually become apparent from the first to the second week of life, whereas the central nervous system (CNS) form typically manifests up to the fourth week, not exceeding the sixth week of life. Most cases (up to 85%) are SEM. At this stage, cerebrospinal fluid analysis and neurological findings are normal. Infected children may be asymptomatic, but when clinical manifestations are present, they may include clustered vesicles on an erythematous base or severe tearing with ocular discomfort and conjunctival hyperemia. Although considered a benign form of the disease, it may leave sequelae, particularly ocular impairment, and progress to the localized CNS form, with symptoms such as lethargy, thermal instability, irritability, altered feeding pattern or poor acceptance, bulging fontanelle, seizures, and lethargy. When the disease progresses to the disseminated form, there is involvement of additional organs such as the brain, lungs, liver, and adrenal glands, which occur in approximately 25% of infected neonates [4].

Thus, the present review aims to compile information and analyze the importance of herpes simplex virus infection during pregnancy, as well as discuss its implications for maternal, fetal, and neonatal health and the clinical management of the disease.

## 2. Methods

This narrative review was conducted to synthesize current evidence on the maternal–fetal implications of HSV infection during pregnancy. A structured literature search was performed in the electronic databases PubMed/MEDLINE, Scopus, and Web of Science, covering studies published from January 1980 to February 2026. The search strategy combined controlled vocabulary (MeSH terms) and free-text keywords, including “herpes simplex virus”, “HSV”, “pregnancy”, “vertical transmission”, “neonatal herpes”, “perinatal outcomes”, “diagnosis”, and “treatment”.

Eligible studies included original research articles, systematic reviews, meta-analyses, and international guidelines addressing HSV infection in pregnancy and its maternal, fetal, and neonatal implications. Articles were selected based on relevance, methodological quality, and contribution to the understanding of epidemiology, pathophysiology, clinical presentation, diagnosis, and management. Reference lists of key publications were manually screened to identify additional relevant studies.

As this is a narrative review, no formal protocol registration, risk of bias assessment, or quantitative synthesis was performed. However, the review was conducted with a structured and comprehensive approach to ensure a balanced and up-to-date synthesis of the available evidence.

## 3. Virological and Immunological Aspects

HSV is an extremely prevalent pathogen in humans, capable of causing various diseases such as herpes labialis, genital herpes, herpetic stromal keratitis, disseminated disease in neonates, meningitis, and encephalitis [5]. These are double-stranded DNA viruses of the Herpesviridae family, which can be differentiated into type 1 and type 2 due to differences in the glycoproteins of their lipid envelope. HSV-1 contains glycoprotein G1 and is responsible for herpes labialis, gingivostomatitis, keratoconjunctivitis, and genital herpes. HSV-2 possesses glycoprotein G2 and, according to current knowledge, is almost always the etiologic agent in cases of genital herpes. The virus is transmitted through direct contact with an infected individual via injured skin or mucosa. The incubation period ranges from 2 to 12 days [6].

In primary infection, the individual may be asymptomatic even while the virus replicates in the dermis and epidermis; however, after the multiplication period, HSV invades local nerve endings and neurites and, through retrograde dissemination, lodges in the ganglia of the peripheral nervous system of its host, in sensory and autonomic ganglia, where it remains viable but in an inactive form, constituting its latency period, which allows later reactivation [5]. In primary infection, the virus enters the body through the mucosa or injured skin and infects epithelial cells. The pathogen possesses protective and immune evasion strategies, including its own structural organization. Its deoxyribonucleic acid (DNA) is enclosed by the capsid and tegument, which protect the genetic material from enzymes, temperature changes, and mechanical impacts. In addition, its envelope is rich in phospholipids acquired from host cells; this evasion mechanism causes the virus to be recognized by the immune system as self. Within the viral lipid envelopes are glycoproteins; these structures project into the extracellular environment, and their presence allows recognition and adhesion to mucosal or epithelial cells and rapid invasion. The main ones are gB, gC, gD, gH/gL, and gG. The two main structures responsible for viral adhesion to cells are gB and gC, known as glycosaminoglycans (GAGs). After binding, protein gD attaches to specific receptors such as Herpesvirus entry mediator (HVEM), nectin-1, nectin-2, and 3-O-sulfated heparan sulfate; this process allows entry into the cell. Each receptor is related to a different infection site; for example, nectin-1 is used for infection in the nervous system, whereas HVEM is the entry route in the cornea. With this initial binding, gD changes its conformation and connects to the gH/gL complex, activating gB. This glycopeptide is responsible for membrane fusion between the cell and the virus. Once inside the cell, the virus is transported by cellular proteins to the nucleus. Finally, the capsid anchors to the nuclear pole and releases its DNA. After invading the nucleus, cellular ribonucleic acid (RNA) polymerase II transcribes HSV genes and triggers its production sequence. The transcriptional cascade begins with immediate genes, which stimulate replication, exert regulatory functions, and activate early genes, allowing the replication of viral DNA and subsequent production of new viral particles. Once genome replication is completed, late genes produce the structures that compose the virus. Once assembled, the virus undergoes an initial envelopment process that allows it to exit the nucleus and remain free in the cytoplasm. There, the capsid becomes free through unfolding, and tegument proteins adhere to its surface. Finally, a second envelope adheres to the pathogen structure in the Golgi complex and completes viral replication in HSV host cells; enclosed in vesicles, the infectious agent is transported through the cytoplasm and released into the extracellular environment. This process causes intense cellular stress that activates apoptosis, or programmed cell death, and generates tissue damage, which explains the epithelial clinical manifestations [7].

Upon conquering the epithelial environment, productive infection ends; at this moment, HSV may reach peripheral nerve endings, structures that can grow, retract, or degenerate when influenced by factors such as inflammatory cytokines and neurotrophic factors [5]. The virus uses active mechanisms to encourage neurite growth. Glycoprotein G binds to nerve growth factor and intensifies its activity, favoring cellular proliferation. In addition, healthy cells release cytokines that repel neurite approximation; with cellular stress, this process becomes less efficient. Finally, the inflammatory process releases IL-17C, which appears to play a role in increasing the density of the new neural network; thus, a new neurobiological architecture is established that approaches the initial site of infection and facilitates colonization. Frequently, HSV fuses its envelope with the axonal membrane, and microtubules transport the tegument and capsid to the cell body. However, due to cell polarity, tegument proteins are important for the initiation of replication, such as VP16 and ICP0, dissociate prematurely from the virus and are lost in the cellular cytoplasm distant from the nucleus, resulting in poorly expressed immediate early genes and neutralization of viral DNA; this mechanism constitutes latency [7].

The main sensory ganglia of the peripheral nervous system are the trigeminal ganglia (TG) after orofacial infection and the dorsal root ganglia (DRG) after genital infection [8]. Even during latency, neurons may exist across various spectra of activity in which the virus is not silenced but also not in full replication. At this time, viral DNA produces latency-associated transcripts (LAT), a marker of latency in humans that maintains the viral genome silenced and prevents apoptosis. The greater their quantity, the more stable the latency. In addition to viral microRNAs, host neuronal microRNAs play an important role in maintaining latency, as they repress the expression of tegument proteins and hinder access to viral chromatin [9].

Thus, during latency, the virus remains viable but with a certain degree of inactivity; however, when the host organism undergoes some type of stress such as hyperthermia, deprivation of trophic factors, or variation in hormonal levels, repressive mechanisms are transiently destabilized due to activation of the c-Jun N-terminal kinase (JNK) pathway, which allows the expression of some viral genes. This activation is divided into two phases. The first is called animation, in which DNA begins to be transcribed again in very small quantities and does not follow the cascade organization of immediate early, early, and late genes of initial replication. In the second phase, the VP16 protein is expressed again and drives the expression of immediate early genes [9]. After reactivation, new viruses are produced and, through anterograde dissemination, reach the skin and mucosa where they manifest clinically in the typical HSV manner [7].

In the obstetric context, during pregnancy, the organism undergoes physiological immunomodulation in which there is regulated activation of innate immunity but with limitation of effector cell responses, focusing on the tolerability of fetal allogenicity. Regarding adaptive immunity, there is also a reduction in the function of cellular responses and predominance of antibody-mediated immune response [10]. This is unfavorable in combating herpes simplex because this modulation involves reduction of responses fundamental to antiviral protection, especially those mediated by cytotoxic T cells, reducing the mother’s ability to suppress viral replication [11].

In this context, reactivation assumes importance because immunological, hormonal, and metabolic alterations inherent to pregnancy may favor episodes of return of viral activity [12]. During pregnancy, asymptomatic or subclinical reactivations may occur, or even those with clinical manifestations, but with colonization of the lower tract; regardless of type, this represents a risk to the newborn delivered vaginally who passes through the colonized canal and encounters the viral load. However, despite the difficulty in suppressing viral activity, during reactivations, circulating antibodies are already present in the immunocompetent maternal organism; these are transferred to the fetus through the placenta, reducing the risk of severe disease. Thus, the worst scenario occurs when infection is acquired during the third trimester, because the pregnant woman does not yet possess maternal antibodies and her cellular immunity is reduced, favoring vertical transmission [13]. Even if antigens are present and B cells produce IgM efficiently, this still does not confer intrauterine protection; in addition to being less specific, IgM does not cross the placental barrier. With maturation of acquired immunity, IgG is produced and, as the only class of immunoglobulins capable of reaching the fetus, ensures its defense. IgG is a protein that has in its structure a Fab antigen-binding fragment and a stable region, the crystallizable fragment (Fc); through this region, placental transfer occurs by the mechanism of transcytosis. In syncytiotrophoblasts, IgG is engulfed by endosomes with acidic pH, which allows activation of FcRn and its binding to the Fc region of the antibody. As it approaches the cellular basal membrane, pH returns to physiological values, and IgG is released into the fetal circulation. Transplacental transfer begins around the 13th week of gestation and increases with gestational age, peaking at birth. Thus, maternal IgG from a seropositive mother is the main protective factor for the newborn against HSV and, although not capable of preventing infection, reduces its severity and tends to maintain localized disease [14].

The molecular mechanisms underlying HSV infection have direct and clinically relevant implications for pregnancy and neonatal outcomes. Viral entry mediated by glycoproteins such as gD and gB, as well as the virus’s ability to evade host immune responses, contribute to higher viral replication and shedding during primary maternal infection, particularly in late pregnancy, when the risk of vertical transmission is greatest. In contrast, established latency within sensory neurons, followed by periodic reactivation, explains the typically lower transmission rates observed in recurrent infections due to the presence of maternal neutralizing antibodies. These virological dynamics are critical for clinical decision-making, as they underpin risk stratification, influence the timing and indication of antiviral suppressive therapy, and guide intrapartum management, including the mode of delivery. Furthermore, the immature neonatal immune system, combined with high viral exposure during delivery, helps explain the spectrum and severity of neonatal HSV disease, particularly in cases of disseminated or central nervous system involvement.

## 4. Current Epidemiology

### 4.1. Global Prevalence of HSV Infection

**Figure 1:**
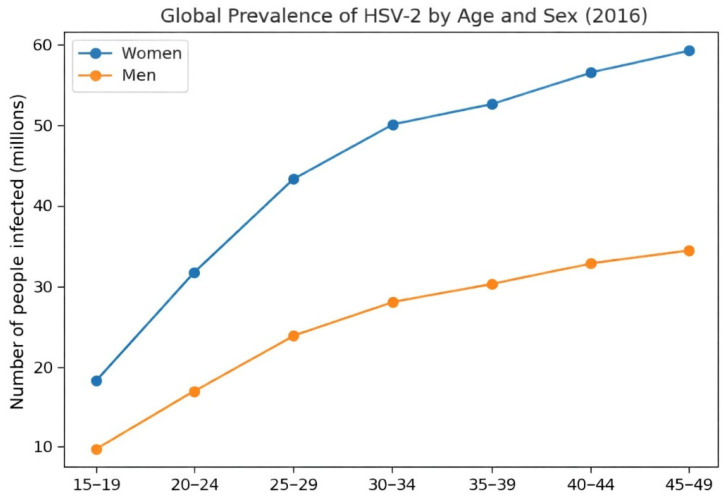
Global prevalence of herpes simplex virus type 2 (HSV-2) infection, by age group and sex, in individuals aged 15 to 49 years in 2016. A progressive increase in prevalence with age and greater involvement of females in all age groups is observed.

In 2020, genital herpes simplex was estimated to represent an important global public health problem. Using a mathematical model based on projections from previous years by the World Health Organization (WHO), researchers inferred that 3.8 billion people worldwide were living with HSV-1, while 520 million had type 2 in that year. Globally, there were 25.6 million new infections in individuals aged 15 to 49 years for type 2, versus 122 million for type 1 in a sample aged 0 to 49 years [15]. In 2016, the global prevalence of type 1 virus was 66.66% and 13.2% for type 2. There is also a sex difference: the prevalence of type 2 disease is almost twice as high in females compared with males in the analyzed regions, namely Africa, the Americas, Southeast Asia, Europe, and the Eastern Mediterranean (Figure 1) [16].

### 4.2. Temporal Trends in Incidence and Seroprevalence

**Figure 2:**
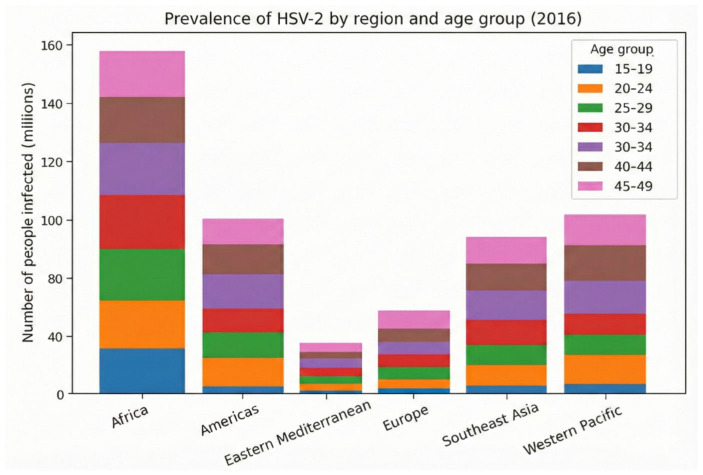
Regional distribution of the global prevalence of herpes simplex virus type 2 (HSV-2) infection, by age group, in individuals aged 15 to 49 years in 2016. Regional heterogeneity and a progressive increase in infection burden with age are observed.

In addition, despite the sex stratification shown in the graph, prevalence increases over the years; however, this does not mean that older individuals have higher chances of infection, but rather that longer lifetime increases the number of risk exposures. Thus, when analyzing age groups, there appears to be an increase in incidence with advancing age; however, these infections are lifelong and cannot be associated with a specific age. In all regions, however, the highest concentration of cases is in the age group between 30 and 34 years. In the regions of Europe and the Western Mediterranean, the absolute number is lower in all age groups; despite the large quantities in the Western Pacific and Southeast Asia, the African continent has the highest number of cases and a relevant contribution in all ages from 25 years onward. In the Americas, there is some difficulty in drawing a direct comparison due to socioeconomic differences, behavioral factors, and heterogeneity of data from each country. This reflects a limitation of the methodology, which in relative evaluation may underestimate or overestimate global data (Figure 2) [16].

**Figure 3:**
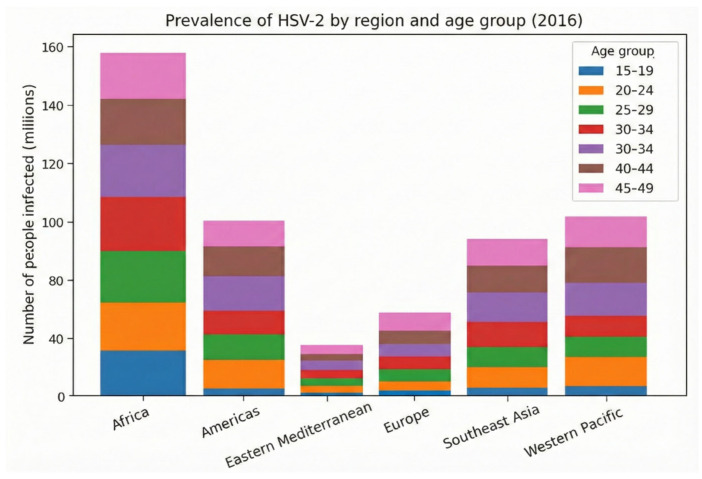
Regional distribution of the prevalence of oral herpes simplex virus type 1 (HSV-1) infection, by age group, in individuals aged 0 to 49 years, in 2016. A high infection burden is observed from childhood, with important regional variations.

**Figure 4:**
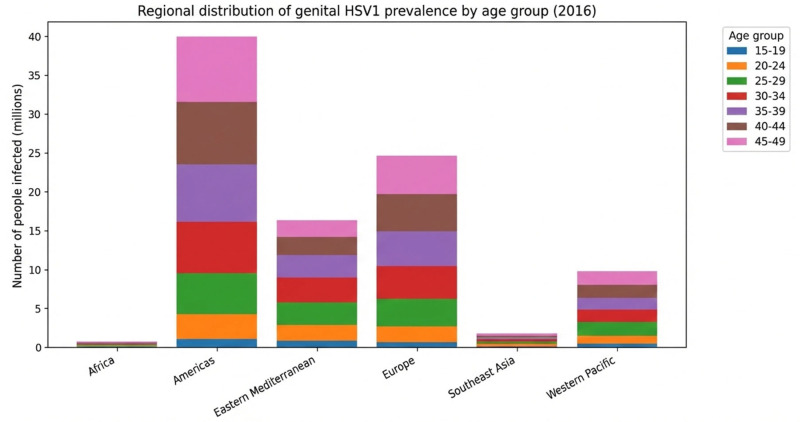
Regional distribution of the prevalence of genital herpes simplex virus type 1 (HSV-1) infection, by age group, in individuals aged 15 to 49 years in 2016.

Bringing this perspective to HSV-1, it is estimated that 3.752 billion people live with the pathogen in the oral or genital anatomical site in the age range from 0 to 49 years, with a prevalence of 66.6%; this number may vary due to double-counting resulting from difficulty in estimating individuals with type 1 infections at both sites. Regarding oral herpes simplex, 3.583 billion people aged 0 to 49 years live with the virus, with a prevalence of approximately 63%; the main affected territories are Southeast Asia and the Western Pacific (Figure 3 and Figure 4) [16].

### 4.3. Evolving Patterns of Transmission, Including Shifts in HSV-1 and HSV-2 Epidemiology

Regarding genital HSV-1, the global prevalence is 5.2%, mainly affecting the Americas and Europe. Compared with the 2012 estimate, an increase in the number of HSV-1 infections is observed, especially with advancing age [16]. This finding denotes a change in the transmission profile of type 1 virus from oral to genital; it was believed that the types rarely affected anatomical sites other than the usual ones; however, evidence indicates that HSV-1 has become the main cause of the first episode of genital herpes. In high-income countries that had a decrease in oral HSV-1 infections, an increase in oral–genital and genital–genital transmission was documented, especially affecting adolescents and young adults, a phenomenon related to exposure and changes in sexual behaviors. It is believed that improvements in hygiene, reduction in family size, and decreased school overcrowding led to a decline in transmission rates in childhood; thus, these individuals did not develop immunity to the virus, and the seroprevalence of the disease decreased. Therefore, when these generations initiated sexual life and had contact with the virus in adulthood, they were susceptible [17]. In the United States of America, it is estimated that up to 25% of all HSV-1 infectious events will occur in the genital tract by 2025. Regarding the disease pattern, it is inferred that in primary infections, viral shedding is frequent and occurs in asymptomatic cases but decreases considerably after the first year due to sustained and early immune response in infected individuals, leading to lower recurrence and clinical manifestations [18].

Although rare, HSV-1 contributes to the increase in the number of neonatal herpes cases and has high morbidity and mortality; this risk is justified because viral shedding in the first months of infection is high, and when it coincides with the gestational period, the greater number of cases related to type 1 virus is understood [18]. Approximately 80% of neonatal herpes cases arise from mothers with unknown disease history. Among women without prior antibodies, approximately 2% acquire HSV during pregnancy. In addition, 10% live with positive partners, that is, they are part of the at-risk population; the obstacle to diagnosis lies in the fact that, even though the disease is the main cause of genital ulcers, a large proportion of cases is asymptomatic. Regarding the pregnant female population known to carry HSV, it is believed that 75% will have recurrences during pregnancy and, among these, 14% have active lesions or other clinical manifestations during the period of delivery [6]. Up to the present moment, this epidemiological pattern has remained stable; however, it is estimated that there will be a reduction in the seroprevalence of pregnant women due to changes in exposure patterns throughout life, mainly related to childhood and changes in sexual behavior, such as fewer partners. This indicates that, in the future, a greater number of women of reproductive age will be vulnerable to primary infection [19]. In summary, HSV has a high global prevalence, affecting diverse regions and age groups; this reflects the dynamic balance between social, behavioral, and biological changes. Recognition of this new scenario is of utmost importance for the development of public health strategies that emphasize prevention and are aligned with trends in sexual and reproductive health and changes in the disease profile.

## 5. Classification of Herpetic Infection in Pregnancy

Herpes infection during pregnancy is a frequent condition and of great importance [4]. HSV is classified as HSV-1, associated with oral infections, and HSV-2, more related to genital herpes and perinatal transmission [20]. Maternal HSV infection may occur in any trimester of pregnancy and is categorized as primary infection (first primary episode), first non-primary episode, and recurrent infection (recurrent episodes) [4,21]. For clarity, primary infection refers to the first HSV infection in a previously seronegative individual, with no pre-existing antibodies to either HSV-1 or HSV-2. A first non-primary episode describes the first clinical episode caused by one HSV type in an individual who already has antibodies to the other type, reflecting partial immunological protection. Recurrent infection refers to reactivation of latent HSV, typically associated with milder clinical manifestations and lower rates of viral shedding compared to primary infection. These distinctions are clinically relevant, as they directly influence the risk of vertical transmission and guide obstetric management.

Primary infection consists of infection by HSV-1 or HSV-2 in a patient previously seronegative for both viral types. It manifests with painful and tender vesicles affecting the external genitalia, vulva, vagina, cervix, and urethra, frequently associated with regional lymphadenopathy and a high risk of vertical transmission (30 to 50%). First nonprimary episode: when the patient presents, for the first time, genital lesions caused by HSV-1 or HSV-2, generally affecting the vulva, in the presence of preexisting antibodies against the virus. Although clinical manifestations are milder compared with the primary infection, in the first primary episode, the risk of vertical transmission may be similar, especially when infection is acquired at the end of pregnancy. Recurrent episodes: correspond to reactivation of a previous genital infection by HSV-1 or HSV-2 and are associated with a lower risk of transmission to the newborn due to the presence of maternal antibodies transferred through the placenta.

Thus, due to the high viral load and absence of protective maternal antibodies, the risk of vertical transmission of HSV is greater when the pregnant woman acquires a primary infection in the third trimester, particularly in the six weeks preceding delivery. In recurrent infections, this risk is lower due to prior maternal immunity and its protective role for the neonate [4,21].

## 6. Vertical Transmission

Intrauterine infection may result from hematogenous spread of the virus after primary maternal infection, especially if significant viremia occurs. Consequences for the fetus include spontaneous abortion, stillbirth, congenital malformations, severe lesions, and ocular and neurological impairment [4,22]. The risk of intrauterine transmission is higher in cases of primary maternal infection during pregnancy, as the mother has not yet developed protective antibodies that could be transferred transplacentally to the fetus [23].

The diagnosis of intrauterine HSV infection is confirmed by detection of the virus in newborn samples [polymerase chain reaction (PCR), viral culture] and by the presence of compatible clinical manifestations [1,4]. The American College of Obstetricians and Gynecologists (ACOG) highlights that intrauterine HSV infections are rare but may occur and recommends special attention to the diagnosis and management of pregnant women with primary infection [6].

### 6.1. Intrapartum Transmission

Vertical transmission of HSV occurs mainly during delivery, when the newborn meets maternal genital secretions containing the virus. Most cases of neonatal herpes result from peripartum exposure (approximately 85%), with intrauterine (5%) or postnatal (10%) transmission being less frequent [24,25]. The risk of vertical transmission is significantly higher when the mother has primary HSV infection at the end of pregnancy, as there is insufficient time for transplacental transfer of neutralizing antibodies to the fetus. In these cases, the risk may reach 30–50% [2,26].

In mothers with recurrent infection, the risk of transmission is much lower (<1%), due to the presence of maternal antibodies that partially protect the newborn [7,8]. Transmission may occur with both HSV-1 and HSV-2, with HSV-1 being associated with a slightly higher risk of vertical transmission [5,7]. Most mothers of newborns with neonatal herpes do not report a history of symptomatic genital lesions, as viral shedding may be asymptomatic [2,26].

Neonatal HSV infection may cause disseminated disease, central nervous system involvement, or localized disease in the skin, eyes, and mouth, with high morbidity and mortality, especially in disseminated cases [24,27,28]. Strategies to reduce risk include suppressive antiviral therapy from the 36th week of gestation in pregnant women with a history of genital herpes and indication of cesarean delivery in the presence of active lesions at the time of birth [29].

### 6.2. Postpartum Transmission

Vertical transmission of HSV occurs predominantly during delivery, but the postpartum (postnatal) period accounts for approximately 10% to 15% of neonatal herpes cases [30,31]. Unlike intrapartum transmission (contact with genital secretions), postnatal infection occurs through direct contact of the newborn with herpetic lesions or infected secretions from caregivers [31]. The mother is the most frequent source, but transmission may occur through other family members or healthcare professionals [32]. Contact usually occurs through oral lesions (herpes labialis), cutaneous lesions on the hands or other parts of the body, and through the nipple (herpetic mastitis) [16,30]. The newborn is particularly vulnerable in the first 4 to 6 weeks of life due to immaturity of the immune system [31].

HSV-1 (generally associated with oral lesions) has become an increasingly more prevalent cause of neonatal herpes [32]. The habit of kissing the newborn by individuals with active lesions (or in a phase of asymptomatic viral shedding) is the main route of contagion [16]. Breastfeeding is not contraindicated if the mother has genital or labial herpes, provided that the labial lesion is covered and hand hygiene is rigorous. However, breastfeeding should be immediately suspended if there are visible herpetic lesions on the nipple or areola [32,33].

### 6.3. Risk Factors

Vertical transmission of HSV may occur in utero (congenital), during delivery (perinatal), or after birth (postnatal). However, approximately 85% to 90% of neonatal infections occur during passage through the infected birth canal [34,35,36]. The risk of transmission is strongly influenced by the mother’s immunological status at the time of delivery. If the pregnant woman acquires HSV for the first time late in pregnancy (third trimester), the risk of transmission to the baby is 30% to 50% [31,36]. This occurs because there has not been sufficient time for production and transfer of protective antibodies (IgG) to the fetus. For women with a history of genital herpes who experience reactivation at delivery, the risk is significantly lower, generally below 3%, due to the presence of preexisting maternal antibodies [35,37].

Factors that increase direct fetal exposure to the virus in the birth canal raise the risk. Such as rupture of amniotic membranes for more than 4 to 6 h before birth, which drastically increases the risk of the virus ascending from the cervix to the fetus [31,38]. Another factor that increases risk is the use of fetal scalp electrodes or forceps, which create microtraumas in the baby’s skin that serve as an entry portal for HSV [36,39]. The presence of visible herpetic lesions or prodromes (pain, tingling) at the time of delivery is a classic risk factor. In these cases, cesarean delivery is recommended to reduce exposure [17,20]. The virus may be shed even without visible lesions. It is estimated that most infected infants are born to mothers without known history or apparent lesions at the time of delivery [38,39].

Although both serotypes cause severe neonatal herpes, HSV-1 (frequently associated with oral infections) has shown increasing rates of genital and neonatal transmission in some regions and is often more aggressive in the newborn central nervous system [19,21]. In addition, pregnant women seropositive for HIV present a greater frequency and duration of HSV shedding, increasing the risk of vertical transmission of both viruses [35,36].

## 7. Impact of Herpetic Infection in Pregnancy

The maternal immune system undergoes functional adaptation during pregnancy, known as immunomodulation. This adaptation is crucial to establish a balance between maternal immunity and fetal development, being necessary to promote and sustain pregnancy and fetal growth [40]. When this balance is disrupted by a viral infection, such as that caused by HSV, homeostasis is broken. This disruption may allow dissemination of the infection and lead to adverse outcomes for both the mother and the conceptus [40]. A recent meta-analysis demonstrated that maternal HSV infection during pregnancy is associated with a significant increase in the risk of adverse perinatal outcomes [41]. Results indicate that infected pregnant women are more likely to experience spontaneous abortion (odds ratio—OR = 3.81), preterm birth (OR = 3.83), and stillbirth (OR = 1.78) [41].

Data from the United Kingdom reveal that 32% of infants diagnosed with neonatal herpes were born premature, a percentage markedly higher than the national average of 7.4% [3]. In addition, clinical outcomes were significantly worse in this group: mortality among preterm infants reached 33%, whereas among term infants it was 19% [42]. The disseminated form of the disease, more frequent in preterm infants, is generally associated with primary maternal infection and presents the highest lethality rate, which increases progressively with decreasing gestational age at birth [42]. Furthermore, the timing of maternal infection proves to be a critical factor for the risk of vertical transmission [43]. During this period, viral shedding tends to be prolonged, and delivery often occurs before the mother has developed neutralizing antibodies capable of protecting the newborn [43]. Approximately 5% of neonatal HSV infections are congenital, resulting from transplacental transmission of the virus. In most cases, approximately 85%In mos occur due to exposure to the virus during delivery, while the remaining 10% are acquired in the postpartum period [44].

## 8. Maternal Clinical Manifestationst

**Figure 5:**
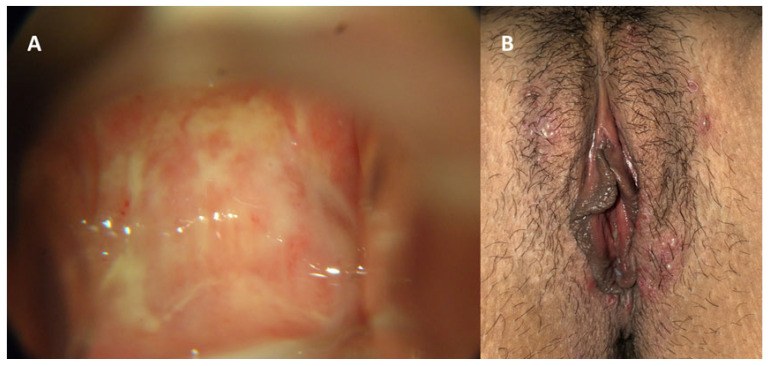
(**A**) Herpetic cervicitis: Colposcopic image of the cervix showing diffuse hyperemia of the cervical epithelium, producing an intensely erythematous appearance. Irregular whitish areas with poorly defined borders are noted, suggestive of fibrinous exudate overlying superficial erosions. Shiny serous secretion partially covers the epithelium, reflecting active inflammation. The cervix appears edematous and friable, with a diffuse inflammatory pattern and no evidence of atypical vascularization suggestive of neoplasia. (**B**) Primary first episode: Multiple bilateral vesiculoulcerative lesions are observed on the labia majora and perineal region. Some lesions still contain translucent vesicular fluid, while others have evolved into shallow ulcers with an erythematous base and irregular borders. Vulvar erythema and edema give the region an inflamed and swollen appearance. Some lesions show a whitish or yellowish base consistent with superficial fibrinous exudate. The multifocal, bilateral distribution is characteristic of primary HSV infection, especially HSV-2 (although HSV-1 may also be involved). The presence of lesions at different stages of evolution (vesicles, erosions, and ulcers), marked inflammation, and extensive involvement is typical of primary infection, which usually presents with more severe clinical manifestations than recurrences.

**Figure 6:**
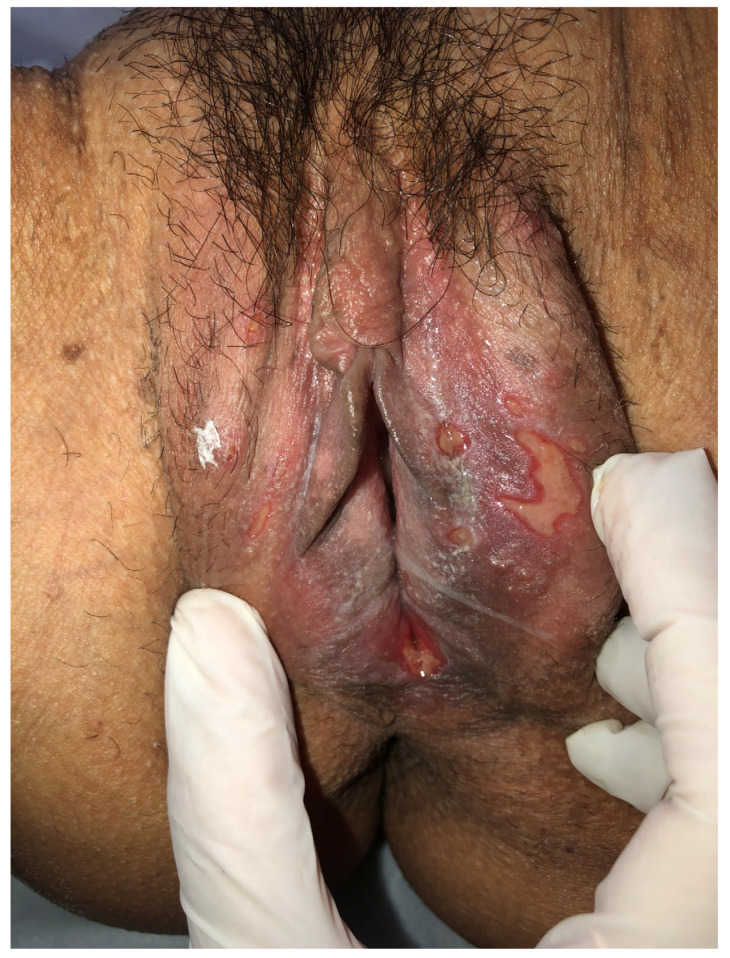
Primary first episode. Marked edema of the labia majora with pronounced diffuse hyperemia and vulvar swelling is observed. Multiple bilateral ulcerative lesions are present, some isolated and others confluent, distributed along the labia majora and perineal region. The ulcers have irregular borders, a peripheral erythematous halo, and a base covered with yellow fibrinous exudate, typical of herpetic ulcerations following vesicle rupture. Intense inflammation gives the mucosa a shiny and friable appearance and may be clinically associated with severe pain, dysuria, and inguinal lymphadenopathy. The multifocal, bilateral ulcerative pattern with exuberant edema is highly suggestive of primary HSV infection, most commonly HSV-2, although HSV-1 may also be implicated.

Genital infection by HSV presents a broad spectrum of clinical manifestations, ranging from asymptomatic conditions to severe and disseminated forms [45]. Symptomatic HSV infection classically manifests as genital herpes and may be categorized into three distinct presentations: primary infection, first nonprimary episode, and recurrent episodes. Primary genital herpes, defined as the patient’s first contact with any viral serotype in the absence of prior antibodies against HSV-1 or HSV-2, is usually the infection of longest duration and greatest morbidity [2]. In these cases, after an incubation period of 2 to 20 days, a severe and prolonged clinical condition develops, which may persist for up to three weeks [1]. Manifestations include multiple and coalescent vesicular and ulcerative lesions affecting the external genitalia, cervix, inner thighs, buttocks, perineum, and perianal region (Figure 5 and Figure 6) [1,45]. Local symptoms are associated, such as intense vulvar pain, dysuria, vaginal discharge, and inguinal lymphadenopathy, in addition to frequent systemic manifestations, including fever, headache, photophobia, and neck stiffness [1,45].

HSV-2 is the main cause of genital ulcer worldwide, being identified in approximately 60% of cases when PCR is used as the diagnostic method [46,47]. Without antiviral treatment, lesions persist for up to three weeks, after which the virus establishes latency in the sacral ganglia, from which it may reactivate periodically [48].

**Figure 7:**
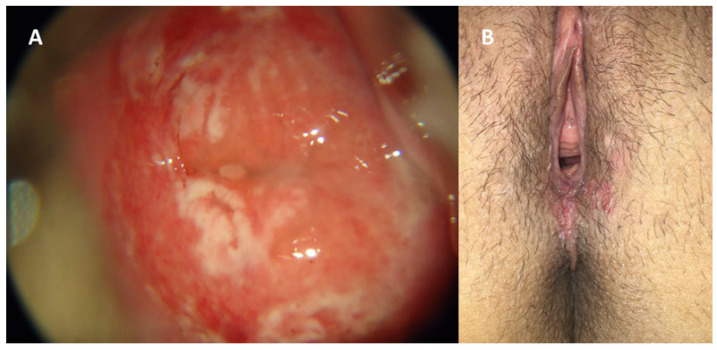
(**A**) Herpetic cervicitis: Colposcopic view of the cervix demonstrating intense diffuse hyperemia of the cervical epithelium. Irregular whitish areas consistent with fibrinous or pseudomembranous exudate overlying the epithelium are observed, associated with multifocal superficial erosions. The lesions have poorly defined contours and an inflamed base, with shiny serous secretion on the surface reflecting active inflammation. Overall, the cervix appears edematous and friable. (**B**) Recurrent episode: Small grouped erythematous lesions are observed in the posterior perineal region and posterior vulvar commissure, some in the stage of superficial erosion. The lesions are well defined, shallow, and small in diameter, with a discrete peripheral erythematous halo and minimal superficial exudate. There is no significant edema of the labia majora, and erythema is localized without notable vulvar swelling. Adjacent skin and mucosa appear relatively preserved, without signs of intense diffuse inflammation. This pattern of grouped, superficial, localized lesions with mild inflammation is typical of herpetic recurrence, generally associated with HSV reactivation, most frequently HSV-2. Clinically, symptoms are usually milder, with less intense pain or burning and shorter duration compared with primary infection.

**Figure 8:**
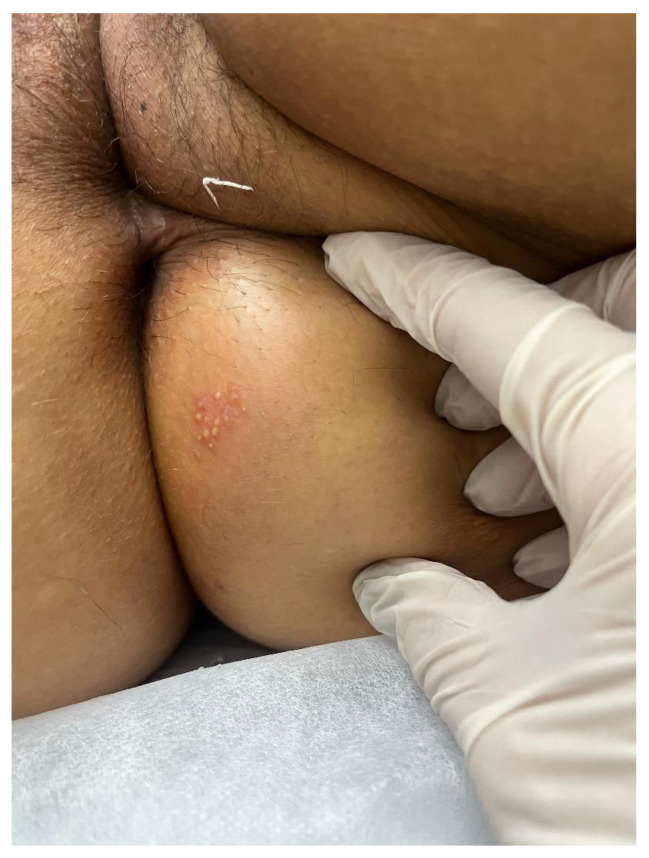
Recurrent episode: A small cluster of vesicles on an erythematous base, forming a typical herpetiform lesion, is observed. The vesicles are millimetric and translucent to slightly opaque. Surrounding erythema is mild and well demarcated, without significant edema of adjacent tissue. There are no signs of extensive inflammation or widespread vulvar involvement, which is characteristic of recurrences, as they tend to be more localized, less painful, and of shorter duration than primary infection. The pattern of grouped vesicles on an erythematous base in an area corresponding to neural reactivation territory is typical of HSV reactivation, usually HSV-2, and may occur in nearby extragenital areas such as the gluteal region due to sacral dermatomal spread.

A characteristic of HSV-2 infection is the presence of recurrences, occurring on average five times in the first year after primary infection, with a tendency toward gradual decrease over time [49]. Although present, recurrences are usually unilateral, milder, and of short duration, frequently preceded by prodromes such as paresthesia, burning, and pruritus in the sacral region (Figure 7 and Figure 8) [45]. It is important to emphasize that manifestations may be atypical, presenting as fissures, localized erythema, or nonspecific symptoms such as headache and low back pain, which frequently lead to delayed or mistaken diagnosis [45]. Another central aspect of HSV-2 infection is the high frequency of asymptomatic viral reactivation, during which viral dissemination occurs in the absence of apparent clinical lesions [50]. One study demonstrated that subclinical shedding occurs on approximately 10.1% of days in asymptomatic individuals and on about 20.1% of days in those with symptomatic infection [50].

Although rare in the general population, disseminated HSV infection is more frequent during pregnancy, particularly in immunocompromised women [51]. The disseminated form presents as encephalitis, hepatitis, disseminated cutaneous lesions, or viral sepsis, in isolation or in association, and is associated with high maternal mortality, reaching approximately 75% when no form of drug therapy was used [51].

In the third trimester, several obstetric conditions present with hepatic abnormalities and should be considered in the differential diagnosis, including HELLP syndrome, preeclampsia, and gestational cholestasis [51]. In these situations, resolution of the maternal condition generally requires termination of pregnancy, unlike herpetic infection, which demands specific antiviral therapy [8]. Additionally, viral hepatitis caused by hepatitis A, B, and C viruses, HIV, Epstein–Barr virus, and cytomegalovirus should be considered as possible diagnoses [51].

**Figure 9:**
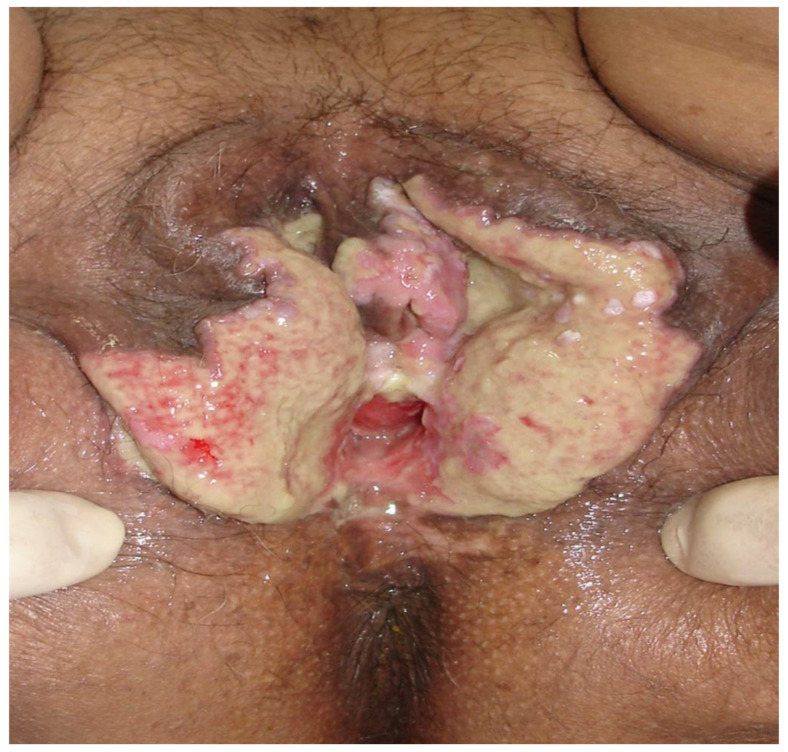
Woman living with HIV: atypical herpetic manifestation: Extensive bilateral vulvar involvement is observed, affecting the labia majora, labia minora, and vaginal introitus. Multiple coalescent, deep, irregular ulcerations are present, with ulcer bases covered by thick yellow fibrinoid exudate, associated with areas of superficial necrosis and devitalized tissue. Lesions have poorly defined erythematous and edematous borders, accompanied by marked vulvar edema with anatomical distortion, superficial hemorrhagic points, and abundant seropurulent discharge extending to the perineal region.

We must also highlight the epidemiological synergy between HSV-2 and HIV: herpetic infection increases the risk of HIV acquisition by approximately threefold and, in people living with HIV, increases the frequency and quantity of HSV shedding, creating a cycle of amplification of transmission of both viruses (Figure 9) [52].

## 9. Diagnosis in Pregnancy

The diagnosis of genital herpes during pregnancy must be confirmed by type-specific laboratory tests. Diagnosis is preferably performed using viral detection techniques, such as viral culture or PCR of active genital lesions (vesicles, ulcers, or other mucocutaneous lesions). PCR is more sensitive than culture, especially in resolving or recurrent lesions. A positive result confirms HSV infection, but a negative result does not exclude the disease, especially in healing lesions [6].

In pregnant women without active lesions but with clinical suspicion or suggestive history, type-specific serologic tests for HSV-1 and HSV-2 may be used. The presence of anti–HSV-2 antibodies is practically diagnostic of genital infection, whereas anti–HSV-1 antibodies may indicate orolabial or genital infection. Distinction between primary and recurrent infection cannot be made solely by clinical signs; it is necessary to combine viral detection and serology (seroconversion or absence of prior antibodies) [6,23].

Routine HSV screening in asymptomatic pregnant women is not recommended by ACOG, as there is no evidence of cost-effectiveness or significant impact on reducing neonatal herpes [1,27,29]. Therefore, diagnosis should be reserved for cases with clinical suspicion or compatible lesions. In summary, the diagnosis of herpes in pregnancy is made by viral culture or PCR of active lesions and, in their absence, by type-specific serology for HSV-1 and HSV-2, as recommended by the ACOG [1,23,29].

### 9.1. Clinical Diagnosis

The diagnosis of HSV infection during pregnancy is one of the most critical pillars of contemporary prenatal care, aiming primarily at the prevention of neonatal herpes, presenting high morbidity and mortality rates [53]. Clinical diagnosis is based on identification of characteristic lesions; however, its sensitivity is limited [54]. Typical manifestations include grouped vesicles on an erythematous base that evolve into painful ulcers, frequently accompanied by painful inguinal lymphadenopathy [55]. The clinical condition is generally more severe in primary infection, with systemic symptoms (fever, malaise). However, primary infection cannot be safely distinguished from nonprimary infection based solely on physical examination [54]. Most transmission occurs from pregnant women without visible lesions at the time of delivery due to asymptomatic viral shedding [53,56].

Although diagnosis is often clinical, based on observation of grouped vesicles, it is insufficient to differentiate viral type and infection status. Limitations of clinical examination include inability to distinguish infections (primary infection and recurrent infection) [6,57], atypical presentations (since, in pregnant women, lesions may be atypical or absent) [54,58], and false negatives (nonspecific symptoms, such as fever or malaise, may mask cases of disseminated herpes, which has high mortality if immediate clinical suspicion is not raised) [6,58].

### 9.2. Laboratory Diagnosis

Due to the often atypical or asymptomatic nature of infection, laboratory confirmation is recommended in all suspected cases [54,56].

#### 9.2.1. Direct Detection of the Virus (Gold Standard)

• PCR: Currently, the method of choice because it is 11% to 71% more sensitive than viral culture [54,56]. It detects viral DNA in lesion swabs (vesicles must be unroofed to collect fluid) [56].
• Viral Culture: Although highly specific, it has lower sensitivity, especially in recurrent or healing lesions. It requires rapid transport to the laboratory [55,56].

PCR is considered the gold standard but is not free from practical limitations, such as collection quality (healing lesions have very low viral load, increasing the risk of false negatives) [54,58], cost and access (it is a high-cost test with limited availability in many primary care centers) [54,57], and viral culture itself, which has lower sensitivity than PCR and takes several days to provide results, making it ineffective for urgent decisions at the time of labor [54].

#### 9.2.2. Type-Specific Serology

• Indication: Used to identify whether the infection is primary or recurrent when the patient has no previous history. It is fundamental for risk stratification [54].
• IgG and IgM: The presence of IgG indicates prior infection (immunity). Seroconversion (changing from IgG negative to positive during pregnancy) or isolated presence of IgM (although less specific) suggests recent infection [53,54].
• Discordant Couples: In 2024/2025, the recommendation to test partners of seronegative pregnant women was reinforced to identify discordant couples and prevent primary infection in the third trimester [54,55].

Serology seeks to identify IgG and IgM antibodies but also presents technical and interpretative challenges, including low specificity, cross-reactivity [54], the immunological window [57], the predictive value of IgM (IgM may reappear during reactivations or persist for months) [58], and routine screening (current guidelines do not recommend universal serologic screening in asymptomatic pregnant women due to high risk of unnecessary anxiety and lack of evidence of clear neonatal outcome benefit for low-risk populations) [6].

## 10. Treatment of Maternal Infection

Management of herpetic infection during pregnancy has the main purposes of attenuating the intensity and duration of the maternal clinical condition, reducing viral shedding, minimizing the risk of vertical transmission, and preventing recurrences in the period close to delivery [4,6,59,60,61].

The three oral antiviral agents used in the treatment of HSV infections are acyclovir, valacyclovir, and famciclovir, indicated for primary genital herpes, recurrent episodes, and daily suppressive therapy, with no evidence of clinical benefit from the use of topical antivirals [4,59,60,62]. Among these therapeutic options, acyclovir is the most used medication in pregnancy, with studies demonstrating a favorable safety profile, including when used in the first trimester, in addition to proven efficacy in reducing viral load and lesion healing time [4,59,61]. Its mechanism of action consists of selective inhibition of viral thymidine kinase, directly interfering with HSV DNA synthesis; valacyclovir, in turn, is a prodrug rapidly converted into acyclovir after hepatic metabolism, presenting similar efficacy and safety but greater oral bioavailability and simpler dosing, which may contribute to better therapeutic adherence, despite higher cost [4,59,61]. Although famciclovir is widely used in international therapeutic regimens, within the context of the Brazilian Unified Health System, it does not constitute a priority antiviral choice, mainly due to its lower availability and higher cost [63]. Pharmacokinetic evaluations in pregnant women have demonstrated the presence of acyclovir in amniotic fluid after maternal administration of acyclovir or valacyclovir, without evidence of selective accumulation in the fetus or increased adverse fetal and neonatal outcomes [4,20].

In cases of first episode herpetic infection, early initiation of oral antiviral therapy is recommended regardless of gestational age, with the objective of reducing symptom duration and viral shedding; treatment may be extended beyond 10 days when complete lesion healing has not occurred, and intravenous acyclovir is indicated in severe or disseminated forms of the disease [4,6]. In situations of recurrent infection, the use of oral antivirals during symptomatic outbreaks is recommended, and for pregnant women with primary, nonprimary infection, or prior clinical history of genital herpes, continuous suppressive therapy from the 36th week of gestation until delivery is indicated, a strategy associated with reduction of clinical recurrences at birth, asymptomatic viral shedding, and the need for cesarean delivery due to active herpetic lesions [4,6,20,63]. It should also be emphasized that, due to increased renal clearance during pregnancy, doses used in suppressive therapy are higher than those used in nonpregnant women, with no consistent reports of relevant maternal adverse outcomes associated with this regimen [20]. Finally, HSV resistance to acyclovir remains uncommon in immunocompetent individuals, being more frequently observed in immunosuppressed patients, which reinforces the effectiveness and safety of the use of these antivirals in the gestational context (Table 1) [64].

## 11. Suppressive Therapy in the Third Trimester

Antiviral prophylaxis in the third trimester is recommended for pregnant women with a history of genital herpes or who have presented primary infection during this period and should be initiated from 36 weeks and maintained until delivery [2,65,66,67,68,69,70]. In cases of primary infection in the third trimester, continuation of treatment until birth may be considered [2,65,66,67,68]. There is no indication for suppressive therapy for women who are only seropositive, without a clinical history of lesions [66,67,68,70].

Recommended regimens include acyclovir 400 mg orally three times daily or valacyclovir 500 mg orally twice daily, starting at 36 weeks until delivery [28,65,67,68,70,71]. Valacyclovir may be advantageous due to its simpler dosing schedule, favoring adherence, although it has a higher cost [65]. Famciclovir is not recommended during pregnancy due to the scarcity of evidence regarding safety [65]. Acyclovir is the antiviral with the greatest scientific support in this scenario and is considered safe for use during pregnancy, including approval by the Food and Drug Administration (FDA) [2,65,66,67].

In the context of delivery, suppressive therapy significantly reduces clinical recurrence (relative risk—RR 0.28) and viral shedding (RR 0.14) [2,28,65,69]. Thus, there is a lower probability of active lesions and viral shedding in the birth canal, which contributes to the reduction of symptoms and duration of lesions, although the preventive effect on episodes throughout pregnancy is limited [70]. Regarding mode of delivery, a reduction in the indication of cesarean delivery for recurrent herpes is observed (RR 0.30) [2,28,65,69,71]. Cesarean delivery is indicated only in the presence of active lesions or prodromal symptoms at the time of delivery, such as vulvar pain or burning suggestive of viral replication [1,2,7,9]. Cesarean delivery is not indicated exclusively due to a history of genital herpes in the absence of clinical manifestations at delivery [65,71].

Regarding the prevention of neonatal herpes, evidence remains limited, as available studies do not have sufficient statistical power to evaluate this outcome conclusively. Although reduction of recurrences and viral shedding suggests potential reduction in the risk of vertical transmission, additional studies are needed to confirm this benefit [28,69]. In general, suppressive therapy in the third trimester is supported by institutions such as the ACOG and the Centers for Disease Control and Prevention (CDC) for pregnant women with a history of genital herpes, to reduce recurrences at the time of delivery and decrease the need for cesarean delivery due to active herpes. Acyclovir and valacyclovir remain the drugs of choice, with well-established regimens and an adequate safety profile during pregnancy.

## 12. Delivery Planning

In women with active genital lesions or symptoms such as burning and tingling, the indicated mode of delivery, according to ACOG, is cesarean section, ideally before rupture of amniotic membranes (however, in the presence of active lesions, it should be performed even if membranes have ruptured) [72,73]. It is known that cesarean delivery contributes to reducing, but does not eliminate, the risk of infection; therefore, even with an asymptomatic newborn, maternal blood should be tested for specific antibodies to determine the type of infection (primary or recurrent), regardless of the chosen mode of delivery [4].

If the patient has a history of genital herpes but presents no active lesions at the time of delivery, there is no mandatory indication for cesarean delivery, since the risk would be greater than the benefit. In addition, in the presence of genital lesions, these should be tested for HSV-1 and HSV-2, and maternal serology should be performed to evaluate whether it is a primary or recurrent infection, given the greater association with vertical infection in primary infection cases.

Although genital herpes is relatively common during pregnancy, there is a scarcity of studies that consistently evaluate the risk of neonatal transmission at the time of delivery and the impact of mode of delivery on this outcome. Available evidence is limited mainly by the small number of cases and, consequently, the low precision of results [74]. In the largest cohort study published to date (2003), Brown et al. [74] analyzed factors associated with neonatal infection in pregnant women with genital herpes. A total of 40,023 participants in the United States were included, of whom 177 (0.4%) had infection confirmed by culture and serology. Among women with primary or nonprimary infection, 17 had vaginal delivery, resulting in seven cases of neonatal herpes (absolute risk 41%), whereas among the nine who underwent cesarean delivery, there was one case (absolute risk 11%). Among pregnant women with recurrent infection, 92 of 151 had vaginal delivery, with two cases of neonatal infection (absolute risk 2%). No cases of neonatal herpes were observed among the 59 women who underwent cesarean delivery in this group. Although the groups are not directly comparable due to the study design, the findings suggest that the probability of vertical transmission is significantly higher in primary infections than in recurrent infections and indicate a protective effect of cesarean delivery in reducing the risk of neonatal infection [74].

Thus, the definition of the mode of delivery should consider the type of maternal infection (primary, nonprimary, or recurrent). In the impossibility of serological confirmation of immunological status, it is recommended to classify the condition as primary infection and adopt the corresponding management [72,74].

## 13. Neonatal Herpes

Neonatal herpes is a severe viral infection acquired during vaginal delivery through contact of the newborn with infected maternal genital secretions [2]. It may also occur, more rarely, through transplacental hematogenous contamination (congenital) or postnatally, through exposure of the infant to cutaneous and oral herpetic lesions [75]. Transmission occurs more frequently in mothers with recent primary infection, particularly in the third trimester, due to high viral load and absence of antibodies transferred to the fetus [2,28,75]. Regarding pathogenesis, most cases are caused by HSV-1, although HSV-2 may be involved, especially in neonatal encephalitis. It is a rare condition; however, it is associated with high morbidity and mortality, especially when not diagnosed and treated early [76,77].

### 13.1. Clinical Forms

Clinical forms of neonatal HSV herpes are classified into three main categories: SEM; CNS; and disseminated multisystem disease. Disease localized to the SEM is characterized by vesicular lesions of the skin or mucous membranes, most commonly in the mouth or eyes. It represents the least severe but most common manifestation of the disease and may progress to more disseminated forms if not treated early [75,77,78,79]. CNS disease occurs in approximately 30% of affected infants and manifests with neurological signs such as irritability, seizures, lethargy, bulging fontanelle, altered level of consciousness, poor feeding, and temperature instability, and it may also present cutaneous vesicles [75]. The disseminated disease form involves multiple organs, including the liver, lungs, adrenals, and CNS. Symptoms are diverse and highly nonspecific, resembling bacterial sepsis, such as temperature instability, lethargy, respiratory failure, hepatitis, coagulopathy, and shock [76,78,80].

### 13.2. Diagnosis

Reliable and rapid diagnostic methods are of great importance, since, as previously mentioned, clinical presentation is nonspecific. Among these, the most sensitive and specific methods are molecular tests, whose samples should be collected as early as possible after symptom onset or perinatal exposure [81,82]. PCR is considered the gold standard for the diagnosis of neonatal infection and has high sensitivity and specificity for cerebrospinal fluid samples in cases of CNS involvement. In addition to cerebral spinal fluid (CSF) analysis, the test detects HSV DNA in samples from skin lesions, mucosa, and blood [83]. In skin and mucosal lesions, PCR may detect the virus even in healed lesions or in situations of low viral load and is considered a faster and more sensitive diagnostic method than viral culture [83,84].

Although it has lower sensitivity and a longer time to obtain results compared with PCR, viral culture is still widely used for the detection of infection in newborns, especially in settings where PCR is not available. In addition, viral culture is more effective when performed from recent vesicular lesions, since viral viability decreases significantly in older or crusted lesions [82,83]. Detection of HSV by PCR in blood may assist in identifying infection, especially in disseminated presentations, whereas PCR in CSF is fundamental in suspected neonatal encephalitis. In addition, blood PCR is recommended in infants exposed to maternal HSV, since the test may be positive even before clinical manifestations begin [85,86].

Regarding serology (IgM and IgG), it should be emphasized that its usefulness is limited for acute diagnosis in newborns, since there is passive transfer of maternal antibodies, in addition to low specificity and sensitivity [82,87]. Furthermore, initial evaluation is extremely important and should include a detailed physical examination with special attention to vesicular lesions, neurological signs, and systemic symptoms. Maternal risk factors such as primary HSV infection, fever, vaginal delivery, and prematurity should be considered [88].

### 13.3. Treatment

Treatment of neonatal herpes varies according to the clinical form of infection. In general, it is based on rigorous clinical support, including intensive care and management of systemic complications, associated with early antiviral therapy with intravenous acyclovir [77,87,89,90,91]. Early initiation of acyclovir therapy markedly reduces mortality from HSV infection: from 85% to 31% in disseminated disease and from 50% to 6% in neonatal encephalitis [87]. Regarding dosage, for a disease limited to the SEM, acyclovir 20 mg/kg IV every 8 h for 14 days is recommended. In neonatal encephalitis and disseminated disease, the dose is the same, but treatment duration is extended to 21 days [77,87,90,91]. Although severe adverse effects of acyclovir are uncommon, renal function and complete blood count should be monitored due to the risk of neutropenia and nephrotoxicity [77,87].

After parenteral treatment, oral suppressive therapy with acyclovir for up to 6 months is indicated to reduce recurrence and improve prognosis, especially in encephalitis and disseminated disease [89].

### 13.4. Prognosis

Prognosis depends on the clinical form and the timeliness of treatment initiation. In general, the SEM form presents a more favorable prognosis; however, delay in initiation of therapy may result in progression to more severe forms. In encephalitis and disseminated disease, even with adequate treatment, high mortality rates and significant risk of neuropsychomotor sequelae are observed [76,77,78,87].

## 14. Prevention and Counseling

Ensuring effective measures to prevent vertical transmission is of utmost importance, given the severity of the disease in neonates. Among these measures are cesarean delivery in mothers with active lesions, avoiding newborn exposure to individuals with active herpes labialis, and implementing suppressive therapy in pregnant women with a history of genital herpes [72]. Suppressive therapy is recommended for all pregnant women with recurrent genital herpes from 36 weeks onward, as it may help reduce recurrence of episodes and, consequently, viral transmission. However, although this therapy may reduce the chance of active genital lesions at delivery, it does not always prevent asymptomatic shedding, so there remains a risk of mother-to-infant transmission [72].

If the virus is acquired in the first or second trimester, oral acyclovir 400 mg three times daily for 5 days is indicated; if infection occurs in the third trimester, the same treatment should be administered and maintained uninterrupted until delivery. It is recommended that if a seronegative woman is in a relationship with a seropositive partner, even if asymptomatic, she should maintain sexual abstinence in the third trimester of pregnancy, including receptive oral sex if the partner presents herpes labialis [4,73,92].

At the first prenatal visit, the patient should be counseled about infections that may affect the fetus or delivery, regardless of herpes diagnosis; however, in serodiscordant couples, if complete abstinence is not accepted, it is essential to recommend selective abstinence during recurrence periods and consistent use of male condoms, which help reduce transmission risk [92]. Furthermore, ongoing research seeks vaccines capable of reducing viral load and preventing primary infection in women before reproductive age. In the past 20 years, three phase 3 human trials tested essential HSV-2 glycoproteins (for viral entry) as immunogens [4]. All showed partial efficacy, such as delay in infection or partial protection in HSV-1/HSV-2 seronegative women against genital HSV-1 infection but failed in the primary objective (prevention of genital disease). Currently, several candidates are advancing, including inactivated HSV vaccines, live attenuated vaccines, replication-defective viruses, protein subunits, and nucleic acids [4].

## 15. Controversial Issues and Future Perspectives

The most relevant discussions regarding HSV infection in pregnancy focus on the utility of universal serologic screening, possible additional preventive approaches, vaccine development, and remaining limitations in scientific production. Universal HSV serology in all pregnant women is a controversial topic. The ACOG and the CDC do not recommend routine serologic testing in asymptomatic women. This recommendation is based mainly on the absence of evidence that screening reduces neonatal herpes incidence, unfavorable cost–benefit ratio, and the potential negative emotional impact of a positive result [29,67,93,94]. Added to this is the fact that many neonatal herpes cases occur in newborns of mothers without prior diagnosis of infection, which reduces the effectiveness of strategies directed only at patients identified as at risk. Although identification of susceptible pregnant women may allow counseling and preventive measures, there is still no solid evidence that such interventions significantly modify neonatal outcomes [95].

In the prevention field, in addition to suppressive antiviral therapy in the third trimester for women with a history of genital herpes, behavioral measures are recommended, such as avoiding unprotected sexual intercourse, especially when the pregnant woman is seronegative, and the partner is seropositive [2,67,93,95]. However, the effectiveness of antiviral use by the male partner with the specific aim of reducing transmission to the pregnant woman remains uncertain [2,95].

Beyond established preventive measures such as suppressive antiviral therapy and delivery planning, additional strategies are increasingly relevant in reducing HSV-related maternal and neonatal morbidity. In serodiscordant couples, particularly when the pregnant woman is HSV-seronegative and the partner is seropositive, counseling on behavioral measures, including abstinence during symptomatic periods and consistent condom use, plays a critical role in preventing primary maternal infection, which carries the highest risk of vertical transmission. From a broader perspective, the development of effective HSV vaccines remains a major unmet need, with ongoing research focusing on both prophylactic and therapeutic candidates targeting viral glycoproteins and immune modulation pathways. Advances in molecular diagnostics, including highly sensitive PCR-based assays, have improved the detection of subclinical viral shedding and neonatal infection, enabling earlier diagnosis and timely initiation of antiviral therapy. Importantly, the burden of HSV infection is disproportionately higher in low- and middle-income countries, where limited access to prenatal care, diagnostic tools, and antiviral medications may increase the risk of adverse outcomes. These disparities highlight the need for context-specific public health strategies, including improved access to screening, education, and perinatal care, to effectively reduce the global impact of HSV in pregnancy.

Regarding immunization, despite continuous research efforts, there is still no approved HSV vaccine. Studies conducted to date have not demonstrated sufficiently consistent results for prevention of primary infection or vertical transmission [29,94]. Several gaps remain in the literature, including the lack of robust clinical trials evaluating the impact of universal screening, the real effectiveness of preventive strategies on neonatal outcomes, and the safety of antivirals in different gestational contexts [94,95]. In addition, studies are needed to explore behavioral and psychosocial interventions, as well as the improvement of diagnostic methods capable of detecting asymptomatic infections with greater precision [94].

## 16. Conclusions

HSV infection constitutes one of the most persistent and prevalent human viral infections, becoming an important public health problem given its ability to establish neuronal latency and reactivate throughout life. HSV-1 is generally associated with orofacial infections and HSV-2 with genital herpes; however, although rare, recent evidence highlights the increasing number of HSV-1 cases as a cause of genital and neonatal herpes. In pregnancy, HSV acquires expanded clinical importance due to immunological changes inherent to this period, which decrease the effectiveness of antiviral cellular response and favor viral replication and reactivation [1,4,20,96].

Neonatal transmission occurs mainly in the intrapartum period but may also occur during pregnancy or postpartum. The clinical presentation of the newborn ranges from localized forms involving skin, eyes, and mouth to severe conditions such as encephalitis and disseminated multisystem disease, responsible for high rates of morbidity, mortality, and neurological sequelae. In this context, early diagnosis, especially by molecular methods such as PCR, together with immediate initiation of intravenous antiviral therapy with acyclovir, is decisive for improvement of neonatal prognosis [5,6,7,8,9,10,11,12,28,75,76,77,78,79,82].

Clinical implications of infection during pregnancy are multiple and require comprehensive management. For adequate risk stratification and decision-making regarding delivery, thorough knowledge of epidemiology, pathophysiology, transmission routes during pregnancy, and clinical presentation is essential. The use of antiviral medications, with emphasis on acyclovir, has been considered safe during pregnancy and effective in reducing viral load. In this context, suppressive therapy from the 36th week of gestation is seen as a fundamental strategy to prevent neonatal herpes, reducing both the number of active lesions at delivery and the need for cesarean delivery [4,61,97].

Although advances exist in knowledge of HSV and in the management of pregnant women and newborns, questions remain. Future research should focus on the development of effective vaccines against viral subtypes to reduce the incidence of primary infection in women of reproductive age. In addition, studies are needed to evaluate screening strategies in high-risk populations to achieve real impact in reducing neonatal herpes. Another important field to be studied is the investigation of molecular mechanisms of latency and viral reactivation, opening the way for new therapies capable of reducing or eliminating viral reservoirs in nerve ganglia.

## Figures and Tables

**Table 1:** Recommended antiviral medication dosages for herpes in pregnancy.

Clinical Indication	Drug	Recommended Dose	Duration/Notes
First episode (primary or non-primary infection)	Acyclovir	400 mg PO, three times daily	7–10 days; may be extended if complete healing has not occurred
	Valacyclovir	1 g PO, twice daily	7–10 days
	Famciclovir	250 mg PO, three times daily	7–10 days
Recurrent episode—episodic therapy	Acyclovir	800 mg PO, twice daily or 800 mg PO, three times daily	5 days or 2 days, respectively
	Valacyclovir	500 mg PO, twice daily or 1 g PO, once daily	3–5 days
	Famciclovir	1 g PO, twice daily or 500 mg followed by 250 mg PO	1 day or 2 days
Suppressive therapy from 36 weeks of gestation	Acyclovir	400 mg PO, three times daily	Start at 36 weeks and continue until delivery
	Valacyclovir	500 mg PO, twice daily	Start at 36 weeks and continue until delivery
Regimens recommended by the Brazilian Unified Health System (PCDT-IST Brazil, 2022) [63]	Acyclovir	200 mg PO, 2 tablets, three times daily	7–10 days
	Acyclovir	200 mg PO, 1 tablet, five times daily	Therapeutic alternative
Suppressive therapy (PCDT-IST Brazil) [63]	Acyclovir	400 mg PO, three times daily	From 36 weeks in selected cases

## Data Availability

No new data were created or analyzed in this study.
